# IP-10 Promotes Blood–Brain Barrier Damage by Inducing Tumor Necrosis Factor Alpha Production in Japanese Encephalitis

**DOI:** 10.3389/fimmu.2018.01148

**Published:** 2018-05-30

**Authors:** Ke Wang, Haili Wang, Wenjuan Lou, Longhuan Ma, Yunchuan Li, Nan Zhang, Chong Wang, Fang Li, Muhammad Awais, Shengbo Cao, Ruiping She, Zhen F. Fu, Min Cui

**Affiliations:** ^1^State Key Laboratory of Agricultural Microbiology, College of Veterinary Medicine, Huazhong Agricultural University, Wuhan, China; ^2^Key Laboratory of Preventive Veterinary Medicine in Hubei Province, The Cooperative Innovation Center for Sustainable Pig Production, Wuhan, China; ^3^Key Laboratory of Development of Veterinary Diagnostic Products, Ministry of Agriculture of the People’s Republic of China, Wuhan, China; ^4^International Research Center for Animal Disease, Ministry of Science and Technology of the People’s Republic of China, Wuhan, China; ^5^College of Veterinary Medicine, China Agricultural University, Beijing, China; ^6^College of Veterinary and Animal Sciences Jhang, Jhang, Pakistan; ^7^Departments of Pathology, College of Veterinary Medicine, University of Georgia, Athens, GA, United States

**Keywords:** blood–brain barrier, IP-10, tumor necrosis factor alpha, tight junction proteins, Japanese encephalitis virus

## Abstract

Japanese encephalitis is a neuropathological disorder caused by Japanese encephalitis virus (JEV), which is characterized by severe pathological neuroinflammation and damage to the blood–brain barrier (BBB). Inflammatory cytokines/chemokines can regulate the expression of tight junction (TJ) proteins and are believed to be a leading cause of BBB disruption, but the specific mechanisms remain unclear. IP-10 is the most abundant chemokine produced in the early stage of JEV infection, but its role in BBB disruption is unknown. The administration of IP-10-neutralizing antibody ameliorated the decrease in TJ proteins and restored BBB integrity in JEV-infected mice. *In vitro* study showed IP-10 and JEV treatment did not directly alter the permeability of the monolayers of endothelial cells. However, IP-10 treatment promoted tumor necrosis factor alpha (TNF-α) production and IP-10-neutralizing antibody significantly reduced the production of TNF-α. Thus, TNF-α could be a downstream cytokine of IP-10, which decreased TJ proteins and damaged BBB integrity. Further study indicated that JEV infection can stimulate upregulation of the IP-10 receptor CXCR3 on astrocytes, resulting in TNF-α production through the JNK-c-Jun signaling pathway. Consequently, TNF-α affected the expression and cellular distribution of TJs in brain microvascular endothelial cells and led to BBB damage during JEV infection. Regarding regulation of the BBB, the IP-10/TNF-α cytokine axis could be considered a potential target for the development of novel therapeutics in BBB-related neurological diseases.

## Introduction

The recent Zika virus (ZIKV) outbreak increased the global interest in mosquito-borne viruses of the genus *Flavivirus*, family *Flaviviridae*. This genus includes Japanese encephalitis virus (JEV), West Nile virus (WNV), Dengue virus, ZIKV, and several other viruses that might cause encephalitis with blood–brain barrier (BBB) disruption (1–3). The presence of *Flavivirus* in the central nervous system (CNS) induces the production of inflammatory cytokines and chemokines, such as interleukin-6 (IL-6), interferon-γ (IFN-γ), C-C motif ligand 2 (CCL2, also known as MCP-1), C-X-C motif chemokine 10 (CXCL10, also known as IP-10), tumor necrosis factor alpha (TNF-α), and IL-8 (4–8). As shown in previous studies, IP-10 is one of the most abundant and earliest chemokines associated with BBB disruption in JEV (7), rabies virus (RABV) (9), and WNV infection (6), but the mechanism through which IP-10 regulates BBB permeability remains unclear.

The BBB is composed of brain microvascular endothelial cells (BMECs), pericytes, and astrocyte endfeet. BMECs directly form the walls of capillaries, whereas pericytes, as contractile cells, regulate the BBB (10–12). Tight junction (TJ) proteins are present among the BMECs, significantly reducing the permeation of polar solutes into the CNS (11). TJs are composed of various protein families, including zonula occludens (ZOs), occludin, and claudins; ZOs link occludin and claudins to the intracellular actin cytoskeleton as scaffolding proteins (13). These TJ proteins seal the interendothelial cleft to form a continuous blood vessel and determine BBB properties (14).

Blood–brain barrier permeability has been reported to be increased in many diseases (15), such as neoplasia, hypertension, experimental allergic encephalomyelitis, trauma, and neurotropic viral infections (14, 16). In an RABV-infected mouse model, TJ proteins (occludin, claudin-5, and ZO-1) are downregulated by cytokines/chemokines, such as IFN-γ, resulting in BBB damage (17). WNV increases the levels of matrix metalloproteinases (MMPs), which degrade TJ proteins and eventually enhance BBB permeability (18). JEV-infected astrocytes release vascular endothelial growth factor, IL-6, and MMP-2/MMP-9, leading to ZO-1 downregulation and disruption of endothelial barrier integrity (19). On the other side, JEV infection leads to microglial activation and subsequent secretion of MCP-1 and TNF-α (20). These complex inflammations are one of the key factors caused neuronal death and eventually resulted in animal death.

As a typical CXC chemokine, IP-10 recruits immune cells such as T cells, NK cells, and macrophages to the inflamed tissue in inflammatory diseases. IP-10 is also considered a biomarker of multiple CNS diseases and is closely correlated with BBB pathological changes (7, 9, 21). Although IP-10 is traditionally recognized for recruiting pathogenic inflammatory cells to inflamed sites, its nonchemotactic role during pathogenesis, particularly its effect on BBB integrity in CNS diseases, remains poorly defined. Growing evidence has shown that IP-10 plays roles beyond immune cell recruitment, including the regulation of activated T cell survival, proliferation, and differentiation (22–24). IP-10 is required for human monocytes to produce a robust range of proinflammatory cytokines in a CXCR3-dependent manner and activate the IκB kinase and p38 mitogen-activated protein kinase (MAPK) signaling pathways (24, 25). The IP-10/CXCR3 axis is generally regarded as a potential therapeutic target in many inflammation-associated diseases (26, 27). IP-10 blockade results in improvements in arthritis and Crohn’s disease (28). These studies suggest that IP-10 might be central in the proinflammatory cytokine network as an inflammation regulator.

Japanese encephalitis virus is a typical neurotropic virus that infects neurons, ultimately leading to severe encephalitis and death (29). Our previous studies have demonstrated that inflammatory cytokines are associated with BBB dysfunction in JEV-infected mice (7). Among these cytokines, IP-10 is highly expressed in the early stage of infection and is central to the cytokine network (7). In the present study, the role of IP-10 in BBB disruption during JEV infection was investigated. Administration of an IP-10-neutralizing antibody significantly alleviated the BBB disruption in JEV-infected mice; however, IP-10 treatment did not alter permeability *in vitro*. It was further found that IP-10 induced TNF-α production through the c-Jun N-terminal kinase (JNK) pathway, and TNF-α modulated the expression and distribution of TJ proteins in endothelial cells, leading to BBB permeability alteration. These observations indicate that IP-10 and TNF-α function as an initiator and an executioner, respectively, in BBB disruption.

## Materials and Methods

### Mice and Viruses

Female C57BL/6 mice aged 6–8 weeks were purchased from the Hubei Provincial Centers for Disease Control and Prevention, Wuhan, China. All mice were raised according to the Committee for Protection, Supervision, and Control of Experiments on Animals guidelines of Huazhong Agricultural University. The JEV-P3 strain has been used in our laboratory previously. For viral proliferation, 5 × 10^4^ plaque-forming units (PFU) of JEV-P3 in 15 µl of Dulbecco’s modified Eagle’s medium (DMEM) were intracerebrally injected into the brains of suckling mice. The mice were sacrificed, and their brains were collected when they were moribund. DMEM at a 10-fold volume of the brain weight was added, and the brains were homogenized on ice. The homogenate was centrifuged at 8,500 r/min for 45 min, and the supernatant was aliquoted and stored at −80°C. The viral titer was determined by plaque formation assays with a baby hamster kidney fibroblast cell line (BHK-21), as previously described (7).

### Cell Lines, Primary Cultures of Glia

Baby hamster kidney BHK-21 [American Type Culture Collection (ATCC)], mouse brain microvascular endothelial bEnd.3 (ATCC), and HEK293T cells were preserved in the laboratory previously. All cell lines were cultured in DMEM supplemented with 10% fetal bovine serum (FBS).

Mouse primary glial cells (astrocytes and microglia) were prepared as previously described (30, 31). Briefly, 1- to 3-day-old newborn C57BL/6 mice were killed by rapid decapitation, and their brains were collected. The meninges were removed, and the cortex parenchyma was cut into pieces in ice-cold Hank’s balanced salt solution containing 0.125% trypsin. The tissues were subsequently digested for 30 min in a 37°C incubator. DMEM (Gibco) supplemented with 10% FBS was added, and the cells were resuspended. The suspension was then centrifuged at 400 × *g* and 4°C for 5 min. The cells were resuspended in complete DMEM (containing 10% FBS) and then cultured in a 37°C incubator containing 5% CO_2_. The next day, the culture supernatant was replaced with fresh complete DMEM. The medium was exchanged every 3 days. After 7–9 days, the cells were shaken at 200 rpm for 24 h. The adherent astrocytes were then cultured in 12-well plate and identified with flow cytometry.

### Flow Cytometry

Confluent monolayers of astrocytes on 12-well plate were digested with 0.25% trypsin, and the cell suspension was collected for flow cytometric analysis. Briefly, after the cells were washed with cold PBS, they were suspended in 0.2% bovine serum albumin (Biosharp) in PBS. The astrocytes were then stained with APC-anti-ACSA-2 (MACS), FITC-anti-CD11b (BD), PE-anti-CXCR3 (BD), or isotype IgG for 25 min at 4°C. The cells were washed twice with PBS and fixed with 1% paraformaldehyde. The populations of astrocytes were detected using a FACSCalibur (BD), and the results were analyzed using CellQUEST Pro software (BD).

### Measurement of BBB Permeability

The mouse BBB permeability was measured with sodium fluorescein dye (NaF, 376 Da) as previously described (32). Six- to eight-week-old female mice were infected with 10^5^ PFUs of JEV-P3 *via* intravenous tail injection. At 5 days postinfection (dpi), the mice received 100 µl of phosphate-buffered saline (pH 7.4) containing 10 mg of NaF *via* intraperitoneal injection under anesthesia. After 10 min, serum was collected, mixed with an equal volume of 15% trichloroacetic acid (TCA), and centrifuged for 10 min at 10,000 × *g*. Then, 120 µl of the supernatant was transferred to a 96-well plate, and 30 µl of 5 M NaOH was added. At the same time, euthanized mice were perfused with cold PBS through the left ventricle of the heart to flush out intravascular fluorescein. The brains were homogenized in cold 7.5% TCA. After centrifugation for 10 min at 10,000 × *g*, 120 µl of supernatant was transferred to 96-well plate supplemented with 30 µl of 5 M NaOH. The fluorescence intensity was measured using Bio-Tek spectrophotometers (Bio-Tek Instruments, Wonooski, VT, USA) with excitation at 485 nm and emission at 530 nm. The brain NaF uptake was normalized to blood fluorescence in at least four animals per group using the following formula: (μg of fluorescence brain/mg of tissue)/(μg of fluorescence serum/ml of blood).

### Neutralization of IP-10 *In Vivo*

Six- to eight-week-old female C57BL/6 mice were infected with 10^5^ PFUs of JEV-P3 viruses by intravenous tail injection on day 0. The mice were then intraperitoneally injected with 100 µl of 1,000 µg/ml IP-10-neutralizing antibody (R&D Systems, Minneapolis, MN, USA) or a rat IgG isotype control (R&D Systems, Minneapolis, MN, USA) on days 0, 2, and 4 postinfection. When the mice were symptomatic at day 5, the BBB permeability was determined based on NaF uptake. At the same time, all brain samples were collected for mRNA or protein detection (*n* ≥ 4 for each group).

### xCELLigence Experiments

Electrode impedance is displayed as a cell indexes (CIs) value, which monitors cell adhesion (33, 34). ACEA xCELLigence real-time cell analyzer (RTCA) Software 2.0 (ACEA Biosciences, USA) was used to measure the CI values. Briefly, bEnd.3 cells were plated on an E-plate 16 at 20,000 cells per well and cultured in a CO_2_ incubator at 37°C for approximately 24 h until the curve became a plateau. The cells were treated with homogenized brain extracts from mock-treated mice (Mock), homogenized brain extracts from infected mice (infected BE), inactivated BE (UV-inactivated infected BE), JEV (multiplicity of infection of 5, 5 MOI), inactivated JEV (UV-inactivated JEV), IP-10 (200 ng/ml; R&D Systems), or TNF-α (100 ng/ml; R&D Systems) as detailed in the figure legends. The viruses in inactivated BE and inactivated JEV were verified by plaque formation assays to ensure that there were no live viral particles. The data are displayed as normalized CI values.

### Transient Transfection and Luciferase Analysis

The TNF-α promoter was prepared by PCR at a specific site in the human genome, and the sequences were then subcloned into the plasmid pGL3-Luc to obtain pGL3-TNF-α-Luc. For transient transfections, HEK 293T cells in 12-well plate were transfected with 0.8 µg of pGL3-TNF-α-Luc or mock transfected (pGL3-Luc plasmid) using Lipofectamine 2000 Reagent (Invitrogen). At 36 h, HEK 293T cells were treated with lipopolysaccharide (1,000 ng/ml) or IP-10 (200 ng/ml). After 12 h, the relative luminescence units were measured using the dual luciferase assay system (Promega).

### Quantitative Real-Time PCR (qRT-PCR) and Enzyme-Linked Immunosorbent Assay (ELISA)

The RNA of mouse brains or cell samples (N2a, bEnd.3, and primary glial cells) was extracted with TRIzol reagent (Invitrogen, Grand Island, NY, USA) and reverse-transcribed into cDNA with a ReverTra Ace qPCR RT kit (Toyobo, Japan) as described by the manufacturer. SYBR Green 2 × mix (Invitrogen) was used for qRT-PCR using a StepOne Plus system with StepOne software v2.2.2 (Applied Biosystems, Foster City, CA, USA). For relative quantification, the mRNA levels were normalized to β-actin expression. The following primer pairs were used for qRT-PCR: TNF-α forward 5′-TCACTGGAGCCTCGAATGTC-3′, TNF-α reverse 5′-GTGAGGAAGGCTGTGCATTG-3′; IP-10 forward 5′-CCTGCTGGGTCTGAGTGGGA-3′, IP-10 reverse 5′-GATAGGCT CGCAGGGATGAT-3′; and β-actin forward 5′-CACTGCCGCATCCTCTTCCTCCC-3′, β-actin reverse 5′-CAATAGTGATGACCTGGCCGT-3′.

Enzyme-linked immunosorbent assay kits (R&D Systems, Minneapolis, MN, USA) were used to measure the expression of IP-10 and TNF-α in mouse brain homogenates and cell culture supernatants according to the manufacturer’s instructions.

### Immunofluorescence (IF)

bEnd.3 cells were treated with infected BE, JEV (5 MOI), IP-10 (200 ng/ml), or TNF-α (100 ng/ml) for 48 h. The cells were then fixed, permeabilized, blocked, and conjugated with primary rabbit anti-occludin polyclonal antibody (pAb; Santa Cruz Biotechnology Inc., Santa Cruz, CA, USA), rabbit anti-claudin-5 polyclonal antibody (Invitrogen), or rabbit anti-ZO-1 polyclonal antibody (Sigma) overnight at 4°C. Goat anti-mouse IgG conjugated with Alexa Fluor 488 was utilized as a secondary antibody, and the cells were then stained with DAPI. The specimens were observed under a laser confocal microscope (Leica, Germany) or a super-resolution structured illumination microscope (SIM; Nikon N-SIM) and analyzed with ImageJ.

### Western Blotting

bEnd.3 cells were cultured in DMEM containing 10% FBS and treated with infected BE, JEV (5 MOI), IP-10 (200 ng/ml), or TNF-α (100 ng/ml) for 48 h. The membrane and cytosol fractions were then prepared with the Mem-PER™ Plus Membrane Protein Extraction Kit (Thermo Fisher Scientific). The mouse brains were lysed in RIPA buffer containing protease inhibitor cocktail (Roche) and phosphatase inhibitor cocktail (Roche), homogenized, and centrifuged at 10,000 × *g* and 4°C for 10 min. The protein concentration of the supernatant was determined with a BCA protein assay kit (Beyotime, China). The protein samples were then electrophoretically separated by sodium dodecyl sulfate polyacrylamide gel electrophoresis at a concentration of 8% (for ZO-1) or 12% (for occludin and claudin-5). The proteins were transferred to 0.22-µm polyvinylidene difluoride membranes (Bio-Rad, Richmond, CA, USA). The membranes were blocked in 5% skim milk in Tris-buffered saline with 0.1% Tween 20 (TBST) for 1 h at room temperature and then incubated with primary rabbit anti-occludin polyclonal antibody (pAb; Santa Cruz Biotechnology Inc., Santa Cruz, CA, USA), rabbit anti-claudin-5 polyclonal antibody (Invitrogen), rabbit anti-ZO-1 polyclonal antibody (Sigma), anti-β-actin monoclonal antibody (Proteintech, China), or JEV-E protein monoclonal antibody (preserved in the laboratory previously) overnight at 4°C. For signaling pathway detection, confluent glial cells were treated with JEV (5 MOI), IP-10 (200 ng/ml), IP-10 (200 ng/ml) combined with IgG isotype (BioLegend; 50 µg/ml), and IP-10 (200 ng/ml) combined with mouse anti-CXCR3-neutralizing antibody (BioLegend; 50 µg/ml) or were subjected to mock treatment (DMEM culture medium) for 1, 2, 4, and 24 h. Rabbit polyclonal antibodies against p38 MAPK, phospho-p38 MAPK-T180/Y182, ERK1/2, phospho-ERK1/2-T202/Y204, nuclear factor κB (NF-κB) p65, LaminA, c-Jun, phospho-c-Jun S63 (ABclonal Technology), JNK, and phospho-JNK-Thr183/Tyr185 (Cell Signaling Technology) were used. The membranes were incubated with horseradish peroxidase-conjugated (HRP) secondary antibody, and the HRP was developed with enhanced chemiluminescence reagents (Beyotime). The data are presented as the means ± SEMs from three independent experiments.

### Nuclear-Cytoplasmic Fractionation

Confluent astrocytes were treated with JEV (5 MOI), IP-10 (200 ng/ml), IP-10 (200 ng/ml) combined with IgG isotype (BioLegend; 50 µg/ml), and IP-10 (200 ng/ml) combined with mouse anti-CXCR3-neutralizing antibody (BioLegend; 50 µg/ml) or were subjected to mock treatment (DMEM culture medium) for 24 h. Nuclear-cytoplasmic fractionation was conducted using the NE-PER Nuclear and Cytoplasmic Extraction Reagents kit (Thermo Fisher Scientific) according to the manufacturer’s protocol.

### Transendothelial Permeability Assay

Transendothelial permeability assays were conducted as previously described with modifications (35). Mouse BMECs (bEnd.3) were cultured on 0.4-µm-pore-size Transwell filters. After the cells reached 100% confluence, control BE, infected BE, 5 MOI of JEV, IP-10, or TNF-α was used to treat the monolayers. Then, FITC-dextran-10000 (10 kDa; Sigma-Aldrich, St. Louis, MO, USA) was applied at 1 mg/ml. The lower chamber medium was collected for fluorescence measurement with a fluorimeter (excitation, 492 nm; emission, 520 nm).

### Ethics Statement

The animal experiments were conducted according to the protocol (number: Hzaumo-2015-018) approved by the Animal Ethics Committee, College of Veterinary Medicine, Huazhong Agricultural University, Hubei, China.

### Statistical Analysis

All experiments were conducted in triplicate. The data are expressed as the means ± SEMs, and the significance of the differences between groups was evaluated by Tukey’s *post hoc* tests. Graphs were plotted and analyzed using GraphPad Prism (v5.0; GraphPad, La Jolla, CA, USA).

## Results

### Neutralizing IP-10 Alleviated BBB Breakdown in JEV-Infected Mice

**Figure 1:**
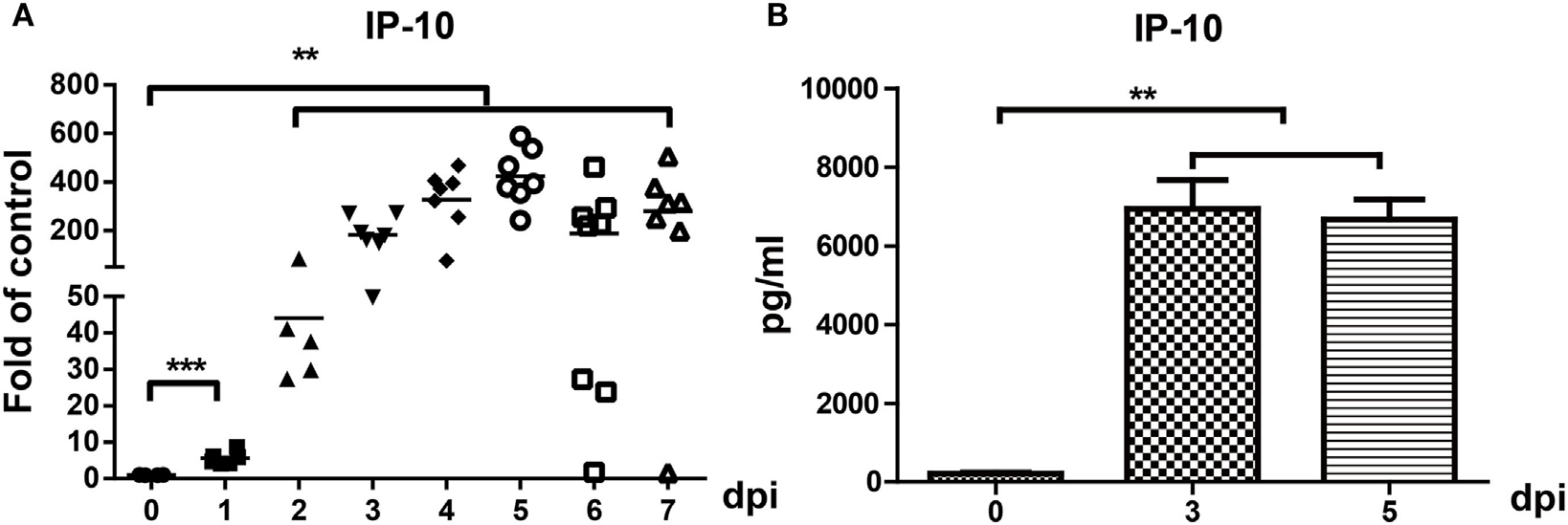
Expression of IP-10 in the central nervous system after Japanese encephalitis virus (JEV) infection. Mice were intravenously injected with 10^5^ plaque-forming units of JEV-P3. **(A)** IP-10 mRNA expression was determined *via* quantitative real-time PCR from 0–7 dpi, and the results show the fold-change of IP-10 relative to the control (*n* = 6). **(B)** IP-10 was detected with enzyme-linked immunosorbent assay on days 0, 3, and 5 (*n* = 4). The results reflect the means ± SEMs from two independent experiments. **p < 0.01; ***p < 0.001.

We previously reported that IP-10 was one of the earliest chemokines produced in the brains of JEV-infected mice. RT-PCR and ELISA were performed to further confirm IP-10 expression in brain tissues. The results showed that the mRNA and protein levels of IP-10 in brains robustly increased from day 1 to day 7 in JEV-infected mice (Figures 1A,B), suggesting that IP-10 might play an important role in JE neuropathogenesis.

**Figure 2:**
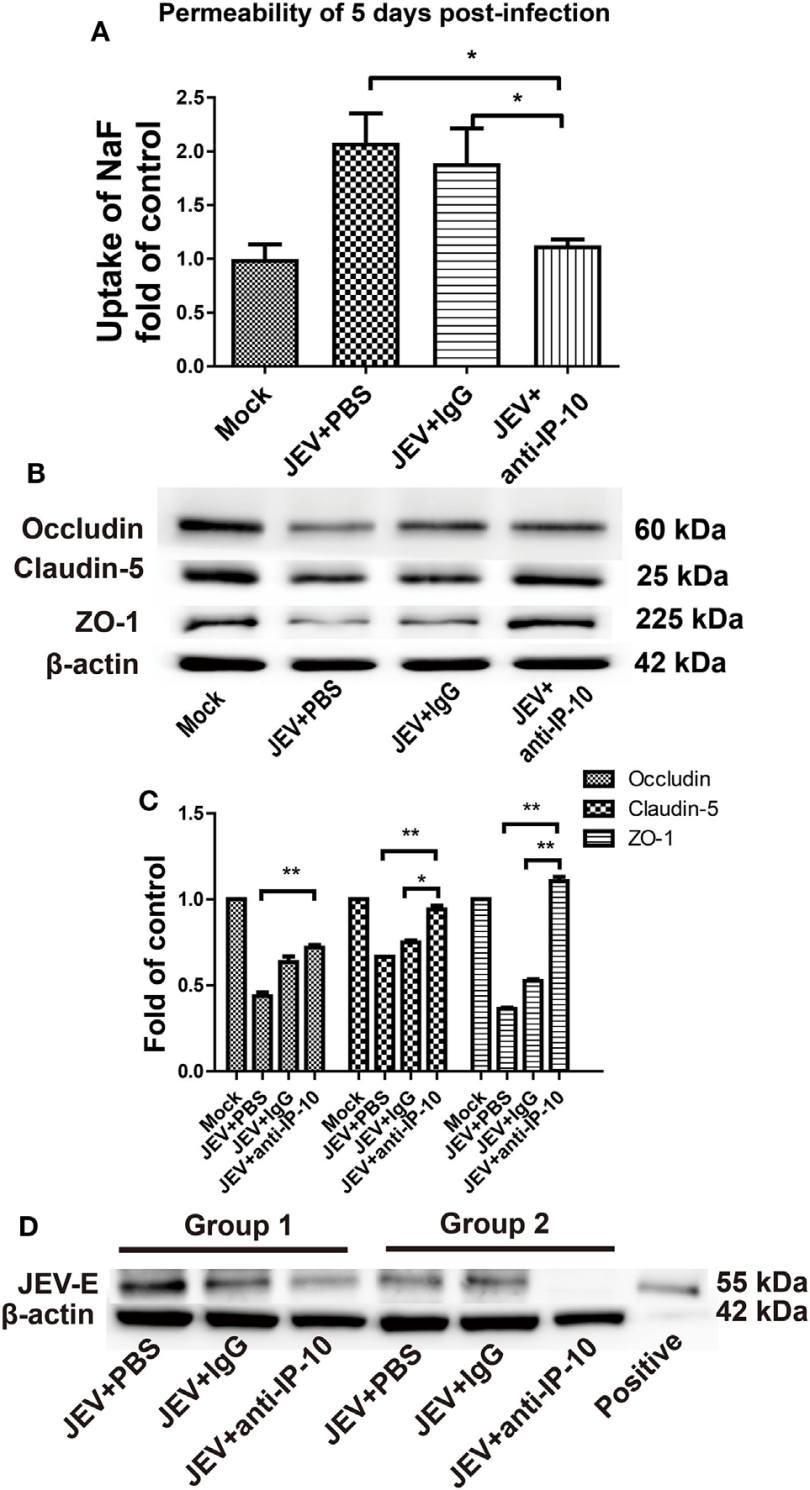
Effects of IP-10-neutralizing antibody on blood–brain barrier permeability during Japanese encephalitis virus (JEV) infection. **(A)** C57BL/6 mice were intravenously infected with 10^5^ plaque-forming units of JEV-P3. On days 0, 2, and 4, the mice were intraperitoneally injected with anti-IP-10-neutralizing antibody, isotype antibody, or PBS. The mice were then sacrificed, and NaF uptake in the brain was measured on day 5 (*n* = 5). **(B)** At the same time, JEV-infected brain samples from the C57BL/6 mice in panel **(A)** were collected, and the expression of tight junction (TJ) proteins were detected by Western blotting. **(C)** The expression of TJ proteins in panel **(B)** were normalized to β-actin expression and quantitatively analyzed as the fold-changes relative to that of the mock-infected controls (*n* ≥ 4). **(D)** The expression of the envelope protein of JEV in mouse brains was measured by Western blotting (*n* ≥ 3). Groups 1 and 2 represent two independent groups of mice. The data are expressed as the means ± SEMs. *p < 0.05; **p < 0.01.

Blood–brain barrier leakage is a typical pathological effect of JE. To investigate whether IP-10 contributes to enhanced BBB permeability, IP-10-neutralizing antibody or isotype controls were intraperitoneally injected into mice on days 0, 2, and 4 after JEV infection. When neuropathological symptoms appeared on day 5, BBB permeability was determined *via* NaF uptake. As shown in Figure 2A, NaF uptake in the control mice (either the PBS group or the isotype controls) was significantly higher than that in the IP-10-neutralizing antibody-treated mice. Compared with the mock-treated mice, no obvious change in BBB permeability was observed in the mice treated with IP-10-neutralizing antibody, suggesting that IP-10 neutralization early in the course of JEV infection could ameliorate the disruption of BBB integrity. To further explore the protective mechanism of IP-10-neutralizing antibody on the integrity of BBB, the expression of TJ proteins, occludin, claudin-5, and ZO-1 were measured by Western blotting. Compared with the mock group, the expression of occludin, claudin-5, and ZO-1 notably decreased in the brains of the JEV-infected mice or the mice infected with JEV and treated with isotype-control antibody. The expression of claudin-5, ZO-1 and, to a lesser degree, occludin, were restored in mice treated with IP-10 antibody (Figures 2B,C). Expression of the envelope protein of JEV (JEV-E) was determined with Western blotting on day 5. Neutralizing IP-10 treatment resulted in decreased JEV-E expression compared with that of the isotype controls (Figure 2D), indicating that IP-10 might affect viral replication. Taken together, our data suggested that IP-10 was a key factor in JE neuropathogenesis and that blocking the IP-10 pathway might protect mice from BBB damage.

### Astrocytes Are the Major Source of IP-10 Production

**Figure 3:**
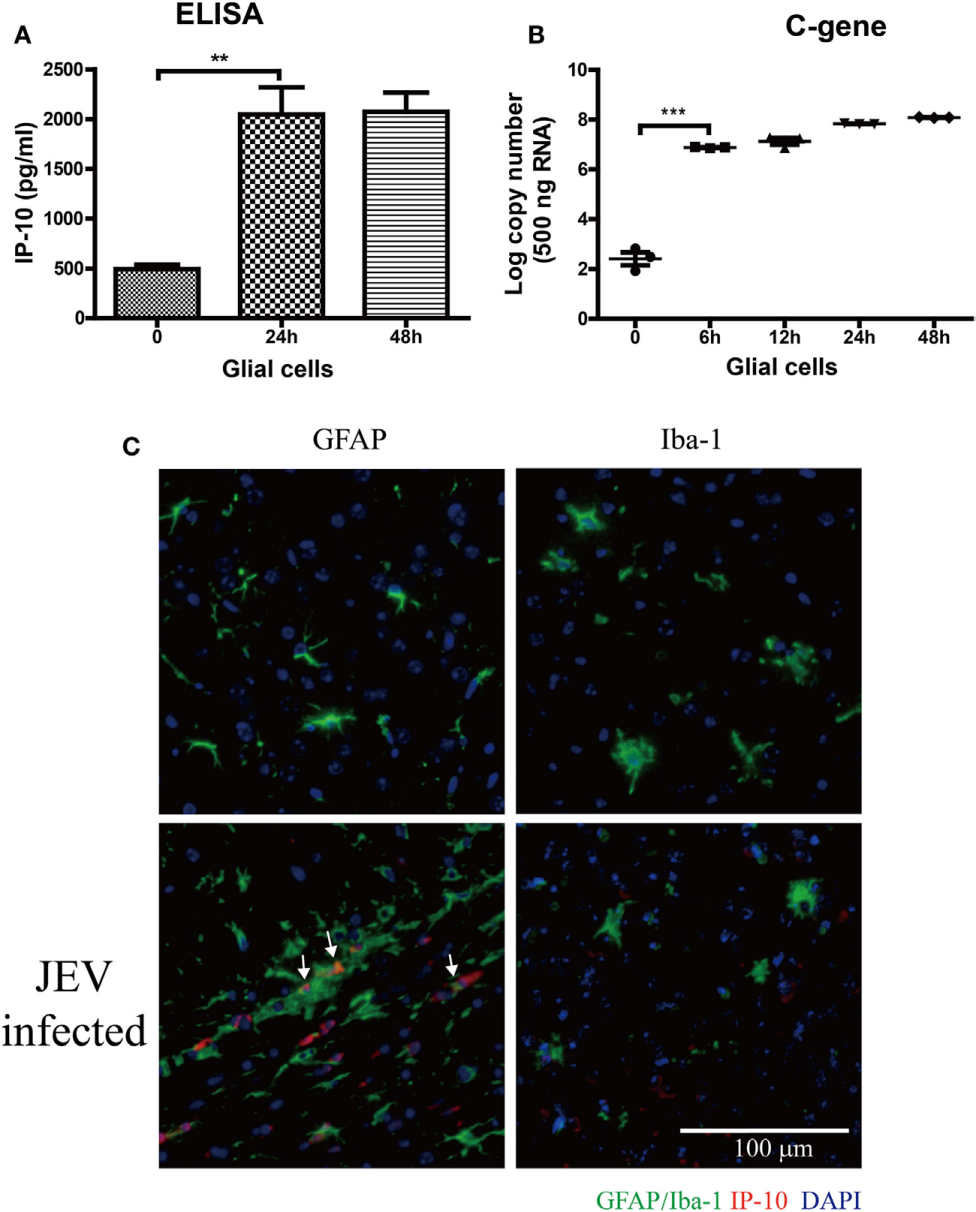
The source of IP-10 on central nervous system. Primary glial cells isolated from suckling mice were cultured in a 37°C incubator containing 5% CO_2_. Then, 5 MOI of Japanese encephalitis virus (JEV)-P3 was added to the well, and mRNA samples were collected. **(A)** IP-10 in the supernatant of glial cells was detected by enzyme-linked immunosorbent assay (ELISA), and **(B)** the copy number of the JEV-P3 C gene in primary glial cells was measured by quantitative real-time PCR. **(C)** Brain sections showing co-staining for GFAP/Iba-1 (green) and IP-10 (red) from mice that infected intravenously with 10^5^ plaque-forming units of JEV-P3 or PBS. Nuclei are shown in blue. White arrow represents the colocalization of GAFP and IP-10. Data are shown as the mean ± SEM (*n* = 3). **p < 0.01; ***p < 0.001.

To determine the source of IP-10 production, the expression of IP-10 was determined on primary glial cells after JEV infection (MOI = 5). IP-10 was significantly increased at 24 h and was stably maintained at 48 h (Figure 3A). Viral RNA was significantly increased at 6 h and reached its peak at 24 h (Figure 3B). Immunostaining showed that astrocytes (GFAP) were the major source of IP-10 (white arrow) rather than microglia (Iba-1, Figure 3C). These data demonstrate that astrocytes were the major source of IP-10 during JEV infection.

### IP-10 Did Not Directly Affect the Permeability of Endothelial Monolayers

**Figure 4:**
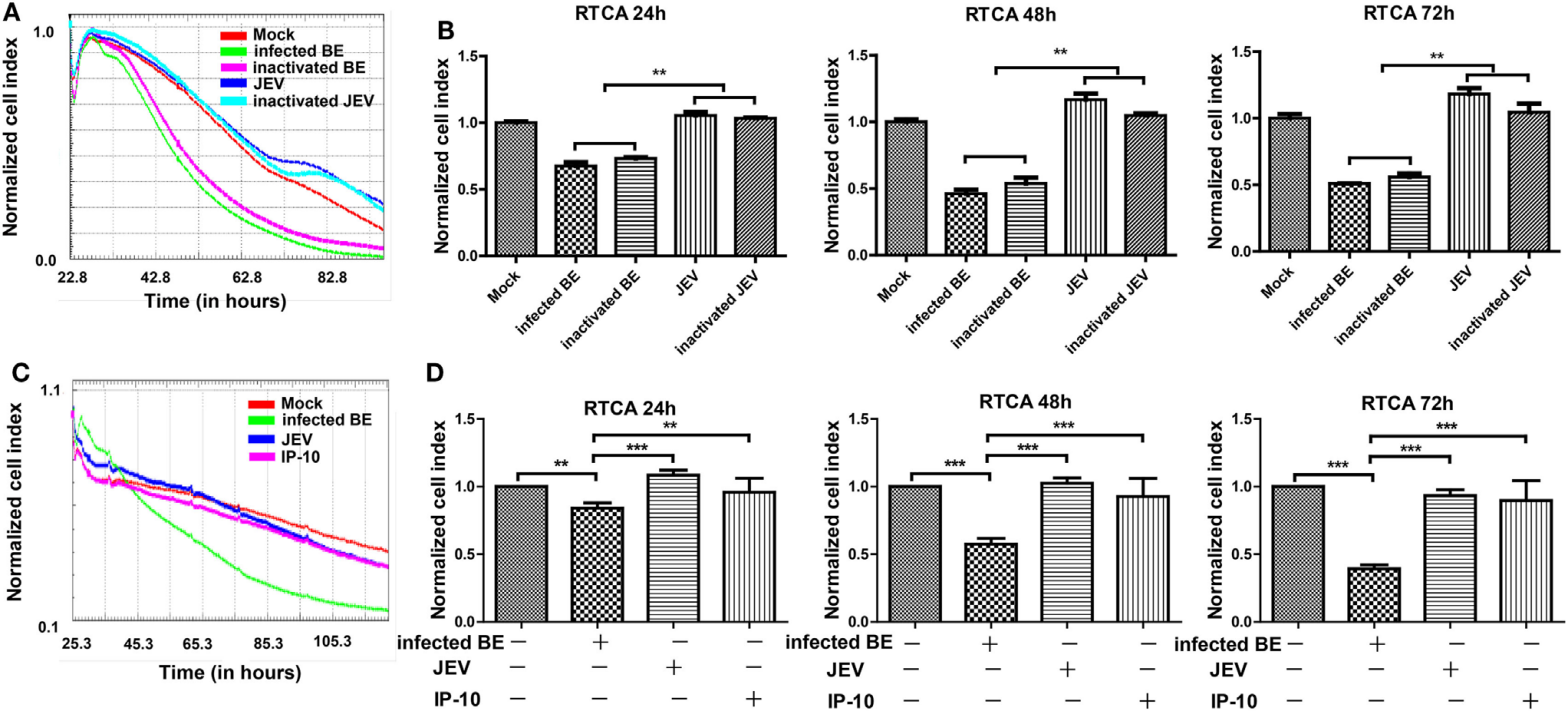
Effects of BE from Japanese encephalitis virus (JEV)-infected mice on bEnd.3 endothelial cells. Monolayers of bEnd.3 cells were treated with infected BE (BE from infected mice), inactivated BE (UV-inactivated BE from infected mice), JEV (5 MOI), inactivated JEV (UV-inactivated JEV), IP-10 (200 ng/ml), or mock treatment (BE from mock-treated mice). **(A,B)** Representative graph of normalized bEnd.3 CIs with different treatments, which were quantified at 24, 48, and 72 h. **(C,D)** Representative graph of normalized bEnd.3 CIs with IP-10 treatment, which were quantified at 24, 48, and 72 h. The results reflect the means of three individual wells ± SEMs. **p < 0.01; ***p < 0.001.

The RTCA is a label-free impedance-based biosensor that is suitable for measuring the impact of drugs on cells (34, 36, 37). When cells were seeded on the RTCA E-plate, data were recorded and exported as the normalized CIs to reflect cell viability, number, and adhesion. To determine whether JEV infection altered BBB permeability directly, we conducted an RTCA experiment on bEnd.3 monolayers. After bEnd.3 cells reached a plateau on an E-plate, the cells were treated with infected BE (brain extracts from JEV-infected mice), inactivated BE (UV-inactivated brain extracts from JEV-infected mice), live JEV, inactivated JEV (UV-inactivated JEV), or mock treatment (brain extracts from mock-infected mice). CIs were real-time monitored with RTCA. The CIs significantly decreased in a time-dependent manner in the cells treated with infected BE (Figures 4A,B). The cells treated with inactivated BE, which contained inactivated JEV particles, showed a trend similar to that observed with infected BE treatment. The CIs showed a lesser decrease in the JEV group and the inactivated JEV group than in the infected BE group. In addition, a few JEV antigens were found in JEV-infected bEnd.3 even infected at an MOI of 10, as previously described (7), indicating that soluble factors, not live viruses, played important roles in endothelial cell permeability.

To further assess whether IP-10 could directly affect BBB integrity, we measured the CIs of bEnd.3 cells using RTCA during IP-10 treatment. After bEnd.3 cells reached a plateau on the E-plate, the cells were treated with infected BE, JEV, or IP-10. Treatment with infected BE substantially decreased the CIs in bEnd.3 cells at 24, 48, and 72 h (Figures 4C,D) compared with the mock group. Compared with treatment with infected BE, IP-10 and JEV had little effect on the CIs of bEnd.3 cells (Figure 4D). All of these data suggested that IP-10 *per se* had no direct impact on the integrity of endothelial monolayers.

### IP-10 Induced TNF-α Expression *In Vivo* and *In Vitro*

**Figure 5:**
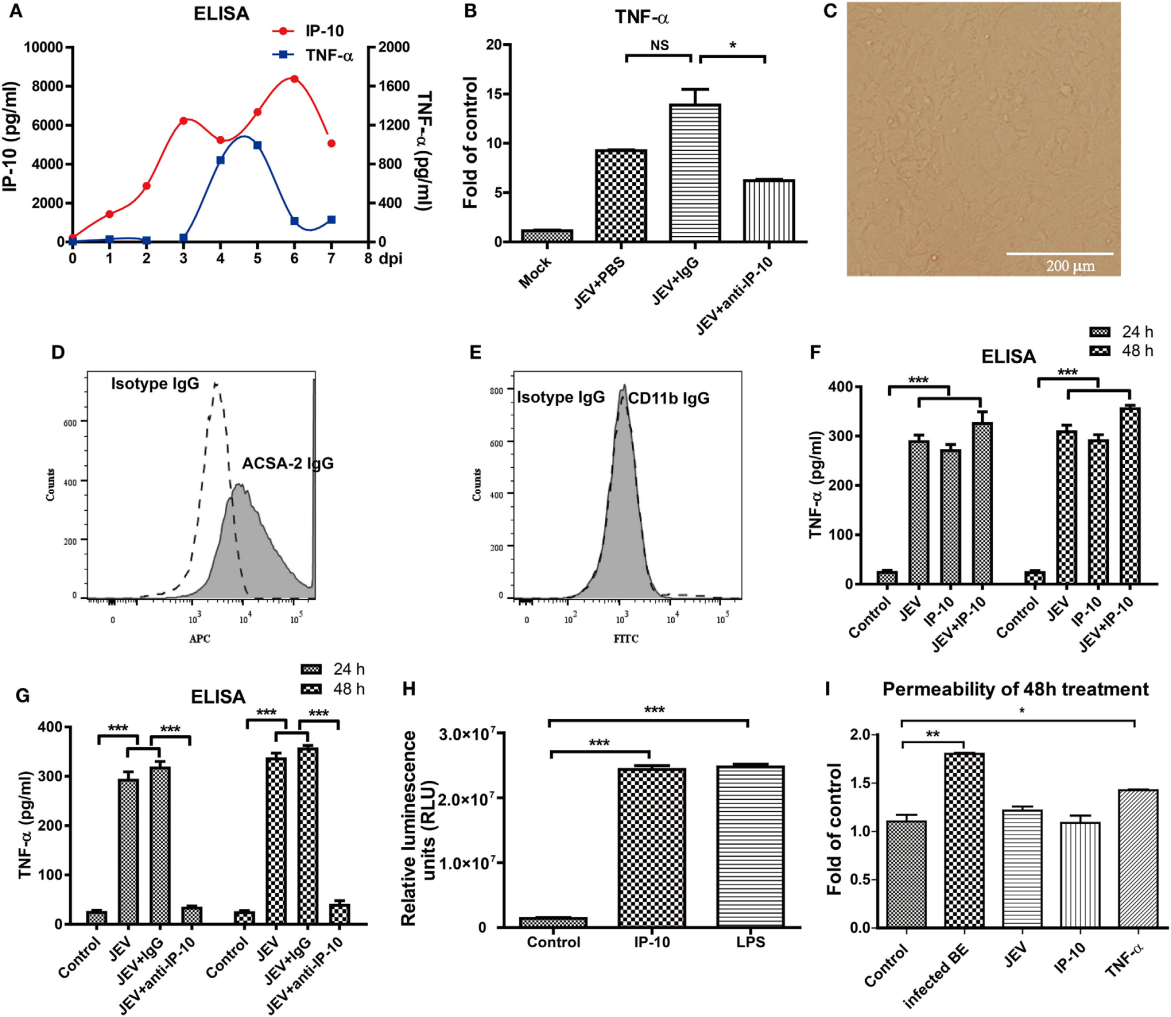
Tumor necrosis factor alpha (TNF-α) expression *in vivo* and *in vitro*. **(A)** IP-10 and TNF-α expression levels in the brain tissues of Japanese encephalitis virus (JEV)-infected mice from day 0 to day 7 were determined by enzyme-linked immunosorbent assay (ELISA) (*n* = 4). **(B)** mRNA level of TNF-α in mouse brains after anti-IP-10-neutralizing antibody, isotype antibody, or PBS treatment. **(C)** The confluent monolayer of astrocytes was observed under a light microscope. **(D,E)** Astrocytes were stained with isotype IgG and IgG against ACSA-2 or CD11b. The populations of astrocytes were detected using a BD FACSCalibur flow cytometer. **(F)** The TNF-α levels in the supernatants of astrocytes treated with IP-10 (200 ng/ml) or JEV at 24 and 48 h were measured with ELISA. **(G)** TNF-α expression in IP-10-neutralizing antibody-treated astrocytes at 24 and 48 h after JEV infection. **(H)** TNF-α luciferase plasmid was transfected into HEK 293T cells, and the luciferase activity was measured after lipopolysaccharide (LPS) (1,000 ng/ml) or IP-10 (200 ng/ml) treatment. **(I)** The transendothelial permeability of bEnd.3 monolayers was measured with dextran-FITC after 48 h of treatment with BE, JEV (5 MOI), IP-10 (200 ng/ml), or TNF-α (100 ng/ml). The data are expressed as the means ± SEMs of the results from three independent experiments. *p < 0.05; **p < 0.01; ***p < 0.001.

Because IP-10 did not change the permeability of the BBB directly and the *in vivo* neutralization of IP-10 prevented JEV-induced BBB damage, IP-10 might influence BBB permeability *via* downstream factors. The relevance of IP-10 and other inflammatory cytokines (TNF-α, IL-6, and IL-1β, which were potentially related to BBB damage) were analyzed in the brains of JEV-infected mice. IP-10 expression increased immediately after JEV infection, 2 days earlier than TNF-α expression (Figure 5A), whereas IL-1β and IL-6 expression levels were significantly increased at 1 and 2 dpi, respectively (data not shown). The RT-PCR results showed that the expression of TNF-α, not IL-6 or IL-1β, was significantly lower after IP-10 neutralization treatment compared with that upon PBS or isotype IgG treatment (Figure 5B). Cultured astrocytes were observed under a light microscope (Figure 5C) and identified with a flow cytometer. After purification, cells were ACSA-2 positive (Figure 5D) and CD11b negative (Figure 5E), indicating that the purified cells were astrocytes. To confirm that IP-10 could induce TNF-α production, primary astrocytes were treated with IP-10 or IP-10 combined with JEV infection. Supernatants were collected for TNF-α detection by ELISA. TNF-α production was significantly increased 24 and 48 h after JEV infection. Interestingly, JEV infection combined with IP-10 treatment significantly enhanced TNF-α expression compared with JEV infection alone at 48 h (Figure 5F). Moreover, IP-10 neutralization treatment significantly decreased TNF-α production in astrocytes (Figure 5G). Furthermore, the luciferase assay specific for TNF-α promoter activity showed that IP-10 could significantly activate the TNF-α promoter (Figure 5H). The transendothelial permeability of bEnd.3 monolayers was measured with FITC-dextran (10 kDa) under different conditions. The results showed that neither JEV nor IP-10 changed the permeability of bEnd.3 monolayers, but TNF-α significantly increased the permeability (Figure 5I). Therefore, these findings indicated that IP-10 could be located upstream of TNF-α and modulate BBB permeability through TNF-α *in vivo*.

### TNF-α Disrupted BBB Integrity by Downregulating TJ Proteins

**Figure 6:**
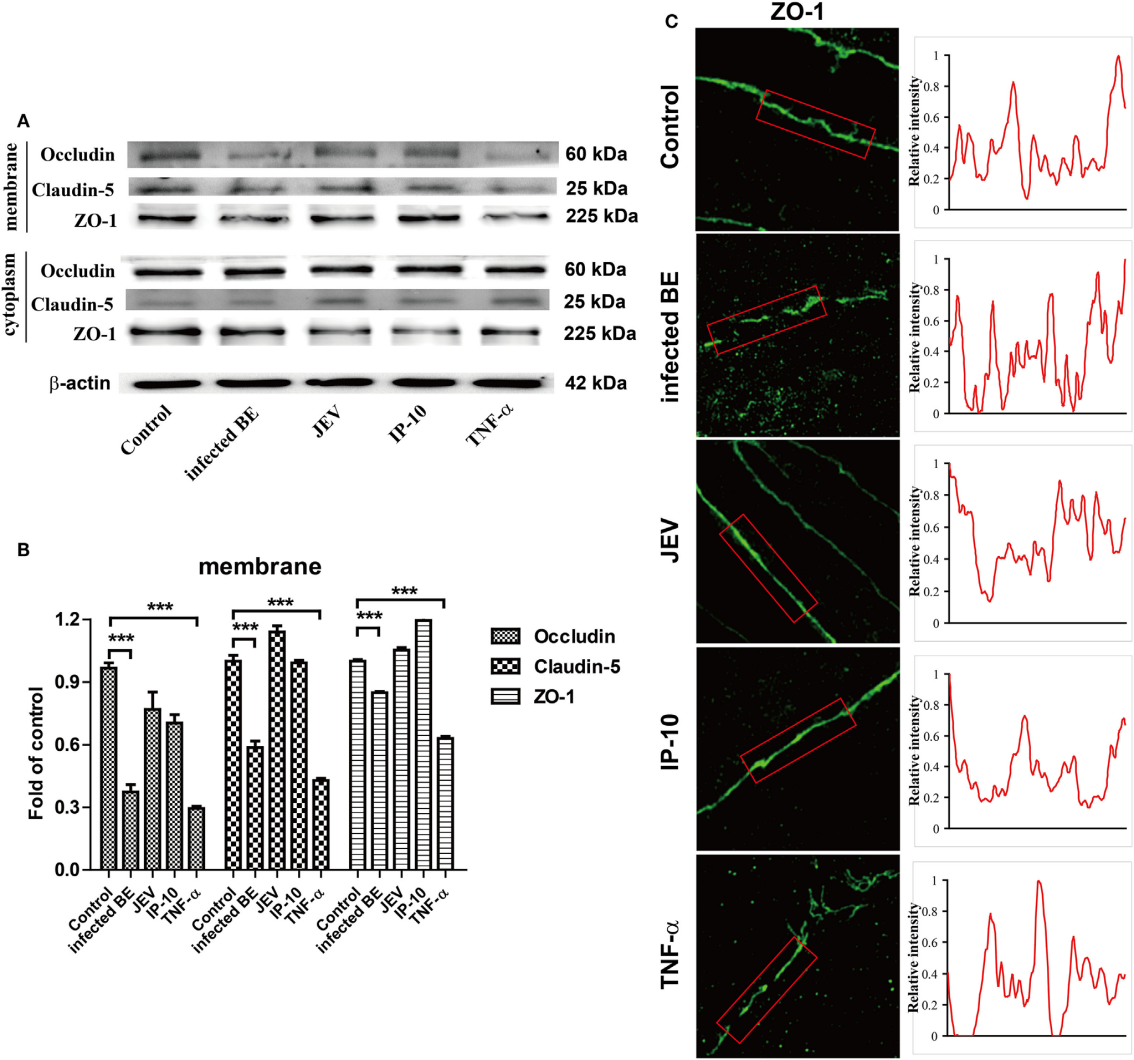
Effects of tumor necrosis factor alpha (TNF-α) on tight junction (TJ) proteins. **(A)** bEnd.3 cells were treated with infected BE, Japanese encephalitis virus (JEV) (5 MOI), IP-10 (200 ng/ml), or TNF-α (100 ng/ml), and the expression of occludin, claudin-5, ZO-1, and β-actin were measured *via* Western blotting at 48 h. **(B)** The expression of TJ proteins in panel **(B)** were normalized to that of β-actin and quantitatively analyzed as the fold-change relative to the control. **(C)** The expression of ZO-1 on bEnd.3 cells after various treatments was detected by super-resolution structured illumination microscope. Relative fluorescence intensities of ZO-1 were obtained on the red box (right panel). Three independent experiments are shown as the means ± SEMs. ***p < 0.001.

Tight junction proteins are important component for the biological function of BBB (7, 19). To further confirm the biofunction of TNF-α on BBB permeability, bEnd.3 monolayers were treated with infected BE, JEV, IP-10 (200 ng/ml), and TNF-α (100 ng/ml) for 48 h, and the expression of TJ proteins were measured by Western blotting. The results showed that TNF-α, rather than IP-10, obviously decreased the expression of occludin, claudin-5, and ZO-1 on cell membranes. Compared with the controls, the reductions in occludin and claudin-5 were approximately 70 and 50%, respectively (Figures 6A,B). We also investigated the distribution of ZO-1 on the cell membranes *via* IF under super-resolution SIM. ZO-1 was exclusively expressed on the membranes of control cells. After 48 h of treatment with infected BE, cell membrane-bound ZO-1 expression was notably decreased and discontinuous, and an increased distribution of ZO-1 in the cytoplasm was also observed. Neither JEV (5 MOI) nor IP-10 (200 ng/ml) treatment changed the cellular distribution and expression pattern of ZO-1 on cell membranes. However, the expression and distribution of ZO-1 with TNF-α (100 ng/ml) treatment showed similar changes to those obtained with infected BE treatment, exhibiting discontinuous expression on cell membranes and trans-cytoplasmic localization (Figure 6C, left panel). Membrane-bound ZO-1 dissociated and clustered after treatment with infected BE or TNF-α compared with that in the controls, indicating that the integrity of the BBB was destroyed. The fluorescence intensity of ZO-1 was quantified with ImageJ software, and the results showed a lower level of fluorescence with TNF-α treatment than in the controls or with JEV or IP-10 treatment (Figure 6C, right panel). These data further demonstrated that TNF-α, not IP-10, damaged BBB integrity by regulating the expression of TJ proteins.

### The JNK-c-Jun Signaling Pathway Was Involved in IP-10-Induced TNF-α Expression

**Figure 7:**
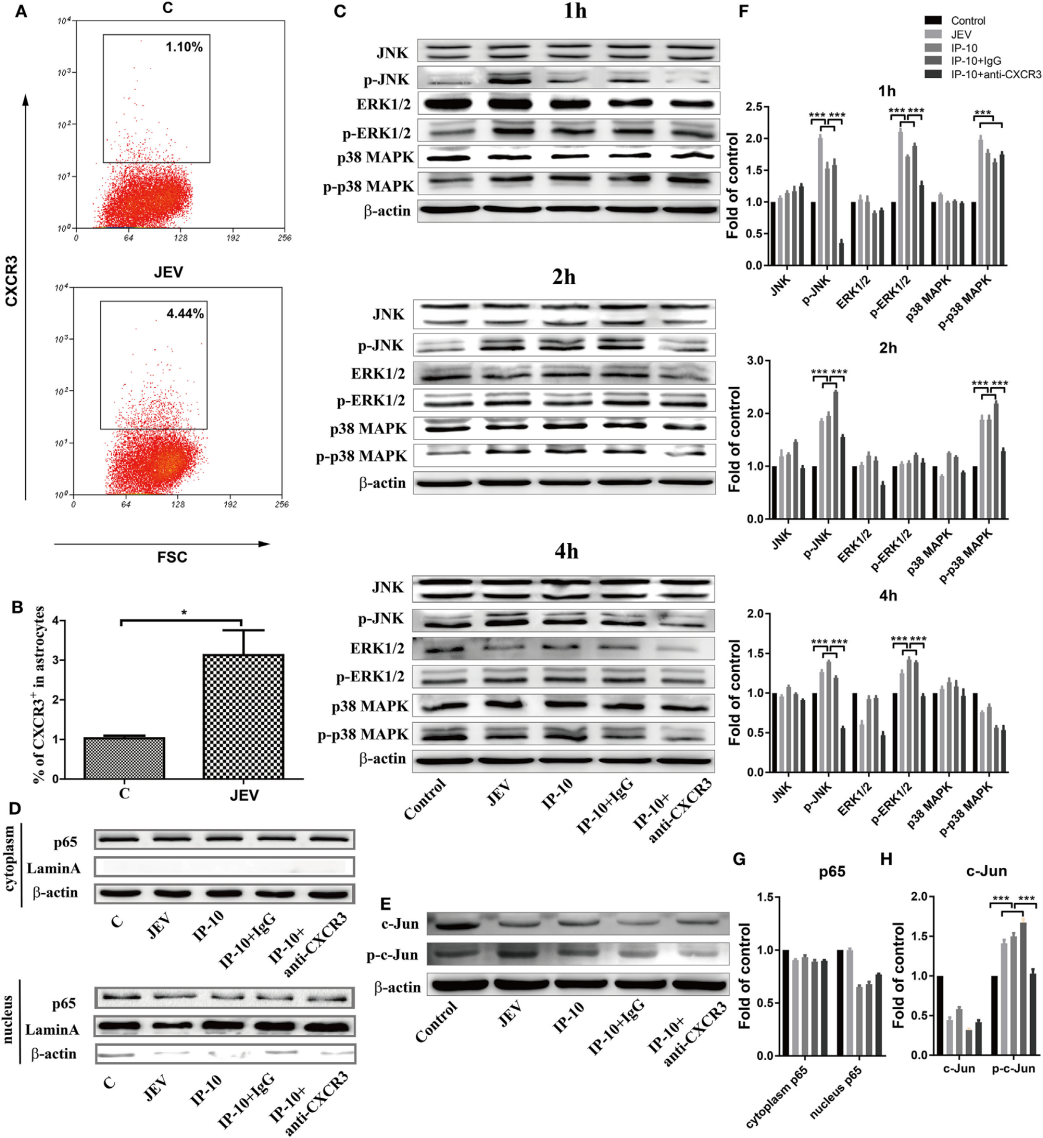
Pathway analysis of Japanese encephalitis virus (JEV) and IP-10 on primary astrocytes. **(A)** Confluent primary astrocytes were treated with Dulbecco’s modified Eagle’s medium (DMEM) **(C)** or JEV (5 MOI). At 24 h postinfection, the cells were digested and analyzed *via* flow cytometry. The data are representative of three independent experiments (gated on live cells). **(B)** Quantification of CXCR3^+^ cells in panel **(A)**. The data are expressed as the means ± SEMs from three independent experiments. **(C)** Western blotting analysis of p38 mitogen-activated protein kinase (MAPK), ERK1/2, and JNK phosphorylation in primary astrocytes. Confluent astrocytes were treated with JEV (5 MOI), IP-10 (200 ng/ml), IP-10 combined with IgG isotype control (50 µg/ml), IP-10 combined with anti-CXCR3 antibody (50 µg/ml), and DMEM (control). The protein samples were collected at 1, 2, and 4 h after treatment, and corresponding antibodies were used to measure the target proteins. The data are representative of three independent experiments. **(D)** The translocation of nuclear factor κB p65 after treatment with JEV, IP-10, IP-10 combined with IgG, IP-10 combined with anti-CXCR3 antibody, and DMEM for 24 h was measured by Western blotting. The data are representative of three independent experiments. **(E)** Transcription factor c-Jun and phosphorylated c-Jun were detected by Western blotting 4 h after treatment. **(F,G,H)** The expression of proteins in panels **(C–E)** were normalized to that of β-actin and quantitatively analyzed as the fold-change relative to the control, respectively. The data are representative of three independent experiments. *p < 0.05; ***p < 0.001.

To further investigate the pathway by which IP-10 induces TNF-α expression after JEV infection, the MAPK signaling pathways, which are closely associated with inflammatory cytokine production (38, 39), were studied in astrocytes. Since CXCR3, a G-protein-coupled protein, is the only receptor of IP-10 (4), its expression profile was determined on primary astrocytes *via* flow cytometry. JEV-infected astrocytes showed higher CXCR3 expression than the controls (Figures 7A,B). Then, the phosphorylation levels of JNK, extracellular signal-regulated protein kinase 1 and 2 (ERK1/2), and p38 MAPK were determined. JEV infection or IP-10 treatment in primary astrocytes enhanced the phosphorylation of JNK at 1, 2, and 4 h, whereas phosphorylated p38 MAPK and phosphorylated ERK1/2 only increased at 1, 2 and 1, 4 h post-treatment, respectively. These results indicated that JEV infection and IP-10/CXCR3 primarily activated the JNK signaling pathway to produce TNF-α (Figures 7C,F). IP-10 blockade with an anti-CXCR3 blocking antibody significantly alleviated this increase, suggesting that IP-10 binding to its receptor CXCR3 activated the JNK signaling pathway. In addition, JEV infection and IP-10 treatment had little or no impact on NF-κB p65 nuclear translocation at 24 h (Figures 7D,G). Furthermore, accumulation of phosphorylated c-Jun was observed in JEV infection or IP-10 treatment, and blocking the CXCR3 signal eliminated this accumulation (Figures 7E,H). Taken together, these results suggested that the JNK-c-Jun signaling pathway was involved in IP-10-mediated TNF-α production.

## Discussion

The BBB is a highly specialized structure that controls the movement of molecules from the periphery to the CNS (40). BBB disruption is a typical pathological effect of JE and other neuroinflammatory diseases, exacerbating neuroinflammation and leading to excessive neuronal damage. Therefore, illuminating the mechanism of JEV-induced BBB breakdown is extremely important for JE therapy and might provide a promising solution for other *Flavivirus* infections and BBB-associated diseases. Inflammatory factors are crucial in BBB disruption. IP-10 is the most highly induced chemokine at the early stage of JEV infection (7); however, it is not known whether IP-10 exerts a direct impact on BBB permeability or activates downstream factors. This report revealed a novel role of IP-10 in the regulation of BBB permeability, which differs from its conventional function and provides potential targets for the development of new therapeutics for BBB-related neurological diseases. In our study, neutralizing IP-10 in JEV-infected mice significantly protected BBB integrity and partially reversed the decrease in TJ proteins. However, IP-10 secreted by astrocytes exerted little effect on BBB integrity. Instead, TNF-α, a downstream factor of IP-10 induced by JNK-c-Jun signaling, directly caused BBB breakdown by regulating the expression of the TJ proteins ZO-1, occludin, and claudin-5. Inhibition of IP-10 markedly decreased TNF-α expression both *in vivo* and *in vitro*, further confirming that IP-10 induced TNF-α influenced BBB permeability.

IP-10 is a key factor in the neuroinflammatory cascade in JE, as reported in our previous publication (7). Temporally, IP-10 production in the CNS occurred prior to BBB damage during JEV infection. However, IP-10 inhibition with neutralizing antibody successfully protected BBB integrity from soluble mediator-induced damage. These data suggested that IP-10 was central in the cytokine network in JEV-induced neuroinflammation. Clear evidence has emerged in recent years to indicate that IP-10 is an initiator of tissue damage in a variety of inflammatory diseases. IP-10 is one of the most abundant proteins detected in the circulation and is also abundantly present in inflamed tissues of patients with inflammation-associated diseases (41). In inflammatory bowel disease, CXCL10 likely leads to intestinal barrier damage by virtue of its abundance and capability to stimulate myeloid-derived cells to produce tissue-damaging cytokines, such as IL-12, IL-23, and TNF-α (24). In a transplantation mouse model, donor islet-specific IP-10 expression has been confirmed to contribute to islet inflammation and loss of β-cell function in islet grafts (42). The effects of islet-derived IP-10 can be blocked by treatment of donor islets and recipient mice with anti-IP-10-neutralizing monoclonal antibody (42). In a Crohn’s disease model, CXCL10 blockade reduces serum IL-12p40, IL-2, IFN-γ, IL-1α, IL-1β, and TNF-α (43). Therefore, IP-10 can act as a CNS inflammatory initiator, affecting BBB integrity directly or indirectly.

*In vitro*, RTCA data demonstrated that IP-10 had no effect on endothelial cells. As the main component of the BBB, BMECs have distinct properties from other tissue cells, which show low CXCR3 expression (undetectable, data not shown). JEV infection in mice significantly upregulated CXCR3 expression on primary astrocytes but not on BMECs. Therefore, biologically, IP-10 can act on astrocytes through CXCR3 rather than the BBB endothelium. CXCR3 is present on astrocytes in human CNS diseases (44). Glia are the major source of inflammatory cytokine in neuropathogenesis (45, 46). CXCR3 was significantly increased on glial cells after JEV infection, and this effect might be associated with the outbreak of inflammation initiated by IP-10 in the early course of JEV infection. Following CXCR3 binding, IP-10 activated the MAPK/JNK signaling pathway. The MAPK family has been found to be involved in inflammation induction in avian influenza virus infection (38) and JEV infection (47). In this study, we showed that JEV-induced IP-10 predominantly activated the JNK signaling pathway and further phosphorylated c-Jun, which is consistent with a previous study that shows that JNK is the primary factor of neuroinflammation in JE (47). Activated JNK and phosphorylated c-Jun are related to prostaglandin E2, TNF-α, IL-1β, and nitric oxide production in glial cells (48, 49). Although NF-κB is a classic trigger of inflammation onset, IP-10-induced TNF-α production was not *via* the NF-κB signaling pathway in JEV infection.

After IP-10 neutralization in JEV-infected mice, cytokine screening with Luminex in brain tissues was performed, including IFN-γ, IL-6, IL-10, IP-10, CCL2, CCL3, CCL4, RANTES, and TNF-α (data not shown). We found that TNF-α showed an obvious corresponding decrease after IP-10 neutralization, indicating that TNF-α might be induced by IP-10. Indeed, IP-10-treated astrocytes produced high levels of the proinflammatory cytokine TNF-α. Once IP-10 was neutralized in JEV infection, TNF-α production was almost completely blocked in astrocytes, suggesting that JEV-induced IP-10 could further mediate TNF-α production in astrocytes.

Tumor necrosis factor alpha is a multifunctional cytokine that regulates various biological processes. A previous study showed that TNF-α is one of the most important inflammatory cytokines in CNS diseases (50). Administration of the anti-TNF-α drug etanercept in JEV-infected mice restores BBB integrity and reduces the viral loads in mouse brains (51), indicating the crucial role of TNF-α in BBB breakdown during JEV infection. TNF-like weak inducer of apoptosis (TWEAK) receptor Fn14 deficiency ameliorates neuropsychiatric disease and reduces CNS inflammation (52). Persistent treatment with TNF-α increases the barrier resistance of endothelial cells (34), and TNF-α promotes angiogenic sprouting in endothelial cells under certain conditions (53), reflecting the mitogenic activity of TNF-α in endothelial cells. Furthermore, TNF-α might regulate the expression and translocation of TJ proteins. The expression of claudin-5 and occludin notably decreased in bEnd.3 endothelial cells after TNF-α treatment. Moreover, along with decreased ZO-1 on plasma membranes, the distribution of ZO-1 clearly became discontinuous. TWEAK, a TNF family member, decreases ZO-1 expression in BMECs and damages BBB permeability *via* the TWEAK/Fn14 pathway (54), which was consistent with our findings. In addition to the decreased expression and discontinuity of ZO-1 on cell membranes, ZO-1 translocation into the cytoplasm increases following TNF-α treatment, and intracellular ZO-1 forms aggregates, indicating dissociation from the cytoskeleton (55).

In summary, the evidence presented in this study demonstrates that IP-10-induced TNF-α mediates BBB breakdown during JEV infection. As a primary cytokine, IP-10 triggers the cytokine cascade in astrocytes, inducing TNF-α production. TNF-α can break down the BBB by reducing the expression of TJ proteins and changing their intracellular locations, thereby damaging the integrity of the TJs between microvascular endothelial cells. Increased inflammatory mediators, including IP-10 and its downstream molecule, TNF-α, compromise the BBB barrier, leading to severe neuroinflammation and neuronal injury. Elucidating the mechanisms of the IP-10/TNF-α axis in inflammation-induced BBB disruption might provide a potential target for therapeutic intervention in BBB-associated CNS diseases.

## Ethics Statement

The animal experiments were conducted according to the protocol (number: Hzaumo-2015-018) approved by the Animal Ethics Committee, College of Veterinary Medicine, Huazhong Agricultural University, Hubei, China.

## Author Contributions

Experimental design: MC, ZF, KW, RS, SC, and YL; performance of experiments: KW, HW, WL, and LM; data analysis: KW, CW, NZ, FL, and MC; manuscript writing: KW, MA, MC, and ZF.

## Conflict of Interest Statement

The authors declare that the research was conducted in the absence of any commercial or financial relationships that could be construed as a potential conflict of interest.

## Abbreviations

BMECs, brain microvascular endothelial cells; Infected BE, homogenized brain extracts from infected mice; Inactivated BE, UV-inactivated infected BE; RTCA, xCELLigence real-time cell analyzer; CI, cell index; dpi, days dpi.
